# The Nanostructured Self-Assembly and Thermoresponsiveness in Water of Amphiphilic Copolymers Carrying Oligoethylene Glycol and Polysiloxane Side Chains

**DOI:** 10.3390/pharmaceutics15061703

**Published:** 2023-06-10

**Authors:** Elisa Guazzelli, Giuseppe Pisano, Marco Turriani, Tarita Biver, Manfred Kriechbaum, Frank Uhlig, Giancarlo Galli, Elisa Martinelli

**Affiliations:** 1 Dipartimento di Chimica e Chimica Industriale, Università di Pisa, 56124 Pisa, Italy; elisa.guazzelli@dcci.unipi.it (E.G.); tarita.biver@unipi.it (T.B.); giancarlo.galli@unipi.it (G.G.); 2 Institute for Inorganic Chemistry, Graz University of Technology, 8010 Graz, Austria; manfred.kriechbaum@tugraz.at (M.K.); frank.uhlig@tugraz.at (F.U.)

**Keywords:** amphiphilic copolymer, self-assembly, polyethylene glycol, polysiloxane, drug delivery, random copolymer, thermoresponsiveness, LCST

## Abstract

Amphiphilic copolymer self-assembly is a straightforward approach to obtain responsive micelles, nanoparticles, and vesicles that are particularly attractive for biomedicine, i.e., for the delivery of functional molecules. Here, amphiphilic copolymers of hydrophobic polysiloxane methacrylate and hydrophilic oligo (ethylene glycol) methyl ether methacrylate with different lengths of oxyethylenic side chains were synthesized via controlled RAFT radical polymerization and characterized both thermally and in solution. In particular, the thermoresponsive and self-assembling behavior of the water-soluble copolymers in water was investigated via complementary techniques such as light transmittance, dynamic light scattering (DLS), and small-angle X-ray scattering (SAXS) measurements. All the copolymers synthesized were thermoresponsive, displaying a cloud point temperature (*T*_cp_) strongly dependent on macromolecular parameters such as the length of the oligo(ethylene glycol) side chains and the content of the SiMA counits, as well as the concentration of the copolymer in water, which is consistent with a lower critical solution temperature (LCST)-type behavior. SAXS analysis revealed that the copolymers formed nanostructures in water below *T*_cp_, whose dimension and shape depended on the content of the hydrophobic components in the copolymer. The hydrodynamic diameter (*D*_h_) determined by DLS increased with the amount of SiMA and the associated morphology at higher SiMA contents was found to be pearl-necklace-micelle-like, composed of connected hydrophobic cores. These novel amphiphilic copolymers were able to modulate thermoresponsiveness in water in a wide range of temperatures, including the physiological temperature, as well as the dimension and shape of their nanostructured assemblies, simply by varying their chemical composition and the length of the hydrophilic side chains.

## 1. Introduction

Amphiphilic polymers are a class of materials that can be employed to obtain self-assembled responsive nanomaterials with great potential in medicine and physiology [1,2]. Even though block copolymers are generally recognized as the workhorse of self-assembled copolymer nanostructures, in recent years, great attention has been directed to the study of the behavior in this field of amphiphilic homopolymers [3,4,5,6] and linear statistical/random copolymers. These materials have the advantage of being accessible via easy, one-step, synthetic procedures such as the straightforward free-radical polymerization [7,8] of hydrophilic and hydrophobic comonomers, but can also display more sophisticated properties when dispersity, chain lengths, and composition are controlled [9,10,11,12] by means of reversible deactivation radical polymerization techniques such as ATRP, RAFT, etc. The variety of structures that can be obtained from self-assembly of these “non-block” amphiphilic copolymer ranges from protein-like folded unimer micelles [13,14,15] and single-chain nanoparticles [16,17,18,19,20] to supramolecular micelles [21,22], which can be characterized by complex internal morphologies such as those shown by necklace micelles [7,23] in lamellar structures [24], large vesicles [25], and micrometric particles [26]. Their unique properties can even show synergistic effects [27] or outperform [28,29] the self-assembly of block copolymers that are generally recognized as the standard for applications where polymer self-assembly is required.

Based on these premises, the rapid development of the field of drug delivery and controlled release took advantage in many cases of the self-assembly of amphiphilic copolymers, and evolved in parallel with the increased understanding of the possible routes to cross multiple biological barriers and allow tissue penetration and intracellular trafficking [30]. In particular, amphiphilic polymeric nanostructure can be exploited as a platform, that can be also functionalized with targeting moieties, in the design of carriers for various payloads, such as nucleic acids, small molecules and proteins, to aid their therapeutic effect and limit their adverse impact ideally targeting specific organs, tissues, or cells [30]. To be effective in such a complex environment, stimuli-responsive self-assembled nanostructures are particularly appealing for biomedical applications such as imaging probes and drug delivery systems susceptible to different triggers, i.e., pH [31], ionic strength [32], polarity [15,33,34], local viscosity and aggregation state [35,36,37], light [9], redox potential [38,39], and temperature [4,40,41,42]. Moreover, the effect of the size of the carriers is one of the more intuitive parameters to be taken into account in the development of more effective carriers. As one example, it was demonstrated that while long circulating drug-loaded polymeric micelles, with diameters of 30, 50, 70, and 100 nm, can all penetrate highly permeable tumors in vivo, particle sizes lower than 50 nm were the most effective to target and to accumulate in poorly permeable tumors [43]. Size also plays a role in conjunction with other nanoparticle characteristics such as particle charge for transcutaneous delivery [44] and surface modification on the cellular uptake with emphasis on the gastrointestinal (GI) barrier and the blood–brain barrier (BBB) [45].

In this study, two series of amphiphilic copolymers with a statistical distribution of hydrophilic oligoethylene glycol methyl ether methacrylate (TEGMA for *n* = 3 or PEGMA for *n* ~ 9) and hydrophobic monomethacryloxypropyl-terminated polydimethylsiloxane (SiMA) were obtained via RAFT copolymerization in a large compositional range (4–65 %mol SiMA). Siloxanes, including polydimethylsiloxane (PDMS), are already in use pharmaceutically both as active pharmaceutical ingredients (API) and as excipients [46], and their use has also been reported in imaging and drug delivery studies due to their good chain flexibility, biocompatibility, and low cytotoxicity [47,48,49]. The thermoresponsive self-assembly of the copolymers in water was investigated via turbidimetry, dynamic light scattering (DLS), and small-angle X-ray scattering (SAXS) measurements. The latter, in particular, revealed that copolymers richer in SiMA aggregated in micelles that showed a tendency to assume a multicore, necklace-like morphology.

## 2. Materials and Methods

### 2.1. Materials

Toluene (Sigma-Aldrich, Darmstadt, Germany) was distilled under vacuum after reflux over calcium hydride. 2,2′-Azobis(2-methylpropionitrile) (AIBN, Sigma-Aldrich, Darmstadt, Germany) was recrystallized from methanol. 2-Cyano-2-propyl benzodithioate (CTA, Sigma-Aldrich, Darmstadt, Germany) was used as received.

Polyethyleneglycol methyl ether methacrylates (PEGMA, *M*_n_ = 475 g/mol, average degree of polymerization ~9, Sigma-Aldrich, Darmstadt, Germany), triethyleneglycol methyl ether methacrylate (TEGMA *M*_n_ = 232 g/mol, Sigma-Aldrich, Darmstadt, Germany), monomethacryloxypropyl-terminated polydimethylsiloxane (SiMA, *M*_n_ = 680 g/mol, average degree of polymerization ~6, Fluorochem, Fluorochem, Hadfield, United Kingdom) were filtered on basic alumina to remove inhibitors. 

### 2.2. Synthesis of Copolymers PEGMA-co-SiMAx

In a typical polymerization, PEGMA (0.78 mL, 1.70 mmol), SiMA (1.21 mL, 1.70 mmol), AIBN (2.22 mg, 0.01 mmol), CTA (15 mg, 0.07 mmol), and toluene (2.2 mL) were added into a Schlenk tube. Three freeze–pump–thaw cycles were performed to remove oxygen; then, the tube was backfilled with nitrogen. The polymerization was carried out at 70 °C. The reaction was quenched by cooling an exposure to air after 15 h. The final product, named PEGMA-*co*-SiMA45, was characterized by ^1^H NMR spectroscopy after purification; x = 45 mol% SiMA. 

^1^H NMR [acetone-d6]: δ (ppm) = 7.4–8 (SC_6_H_5_), 3.9–4.3 (COOCH_2_), 3.5–3.8 (OCH_2_), 3.4 (OCH_3_), 0.8–2.3 (CH_2_CH_2_CH_2_, CH_2_CH_2_CH_3_, CH_2_CCH_3_), 0.6 (CH_2_CH_2_Si), 0.1 (CH_3_Si).

The reaction conditions for the preparation of the other PEGMA-*co*-SiMAx copolymers are summarized in Table S1.

The purification procedure was different for copolymers with different SiMA contents because of the different solubilities. The crude products PEGMA-*co*-SiMA10 and PEGMA-*co*-SiMA17 were precipitated three times from dichloromethane solutions into *n*-hexane; the crude product PEGMA-*co*-SiMA29 and PEGMA-*co*-SiMA45 were purified by dialysis of different copolymer solutions (~0.2 g mL^−1^) against water, water/THF (1/1 *v*/*v*) and THF in the order, using a dialysis membrane (Repligen (Boston, MA, USA) Spectra/Por Biotech RC, 3.5–5 kD cut-off, 16 mm). After dialysis, the copolymers were recovered from the THF solution by evaporating the solvent under vacuum. 

### 2.3. Synthesis of Copolymers TEGMAx-co-SiMAy

In a typical polymerization, TEGMA (0.5 mL, 2.18 mmol), SiMA (1.56 mL, 2.18 mmol), AIBN (3 mg, 0.02 mmol), CTA (20 mg, 0.09 mmol) and toluene (3.1 mL) were added in a Schlenk tube. Three freeze-pump-thaw cycles were performed to remove oxygen then the tube was backfilled with nitrogen. The polymerization was carried out at 70 °C. The reaction was quenched by cooling and exposure to air after 15 h. The crude product was purified by three precipitations from dichloromethane solutions into ethanol (yield 65%). The final product, named TEGMA-*co*-SiMA48, was characterized via ^1^H NMR spectroscopy; x = 48 mol% SiMA. 

^1^H NMR [acetone-d6]: δ (ppm) = 7.4–8 (SC_6_H_5_), 3.9–4.3 (COOCH_2_), 3.5–3.8 (OCH_2_), 3.4 (OCH_3_), 0.8–2.3 (CH_2_CH_2_CH_2_, CH_2_CH_2_CH_3_, CH_2_CCH_3_), 0.6 (CH_2_CH_2_Si), 0.1(CH_3_Si).

The reaction conditions for the preparation of the other TEGMA-*co*-SiMAx copolymers are summarized in Table S1.

The purification procedure was different for copolymers with different SiMA contents because of the different solubilities. The crude products of TEGMA-*co*-SiMA4 and TEGMA-*co*-SiMA6 were precipitated three times from dichloromethane solutions into *n*-hexane. TEGMA-*co*-SiMA65 was precipitated three times from dichloromethane solutions into ethanol. TEGMA-*co*-SiMA15, TEGMA-*co*-SiMA19 and TEGMA-*co*-SiMA28 were purified by dialysis of different copolymer solutions (~0.2 g mL^−1^) against water, water/THF (1/1 *v*/*v*), and THF in that order, using a dialysis membrane (Repligen (Boston, MA, USA) Spectra/Por Biotech RC, 3.5–5 kD cut-off, 16 mm). After dialysis, the copolymers were recovered from the THF solution by evaporating the solvent under vacuum.

### 2.4. Characterization

^1^H measurements were carried out on a Bruker Avance 400 (400 MHz, Billerica, MA, USA) spectrometer with deuterated solvents at room temperature. The sample concentration was approximately 30 g L^−1^. Gel permeation chromatography (GPC) was carried out using a Jasco (Hachioji-shi, Tokyo, Japan) PU-2089 Plus liquid chromatograph equipped with two PL gel 5 µm mixed-D columns, a Jasco RI-2031 Plus refractive index detector, and a Jasco (Hachioji-shi, Tokyo, Japan) UV-2077 Plus UV/vis detector. Samples were filtered via a 0.2 µm PTFE filter before injection. The measurements of number and weight average molecular weights and dispersity (*M*_n_, *M*_w_, *Đ*), relative to PMMA narrow standards, were obtained from refractive index detector curves, in chloroform as the mobile phase (1 mL min^−1^) at 30 °C, maintained by a Jasco (Hachioji-shi, Tokyo, Japan) CO 2063 Plus column thermostat. 

Differential scanning calorimetry (DSC) analysis was performed with a Mettler (Columbus, OH, USA) DSC 922e Module from −130 to 100 °C at heating/cooling rate of 10 °C min^−1^ under a dry nitrogen flow. The glass transition temperature (*T*_g_) was taken as the inflection temperature in the second heating cycle.

A Shimadzu (Kyoto, Japan) 2450 UV–vis spectrophotometer was used for measuring of transmittance of solutions at a fixed wavelength of 700 nm as a function of temperature, in quartz cuvettes with a 10 mm optical path. The cloud point temperature (*T*_cp_) was defined at the middle of the signal drop. UV–vis spectra (200–800 nm) were acquired using a PerkinElmer (Waltham, MA, USA) Lambda 650 spectrophotometer. Solutions in solvents of different polarities were analyzed in a concentration range of 10^−3^–10^−4^ M in quartz cuvettes with an optical path of 10 mm. 

A Malvern (Malvern, Worcestershire, UK) Zetasizer Nanoparticle analyzer (detection angle = 173°) or a Beckman Coulter (Brea, CA, USA) Delsa Nano C particle analyzer (detection angle = 166°) were used for dynamic light scattering (DLS) measurements of the prefiltered copolymer solutions (5 μm PTFE or cellulose acetate filters to reduce contaminant interferences). Intensity, volume, and number distributions from CONTIN analysis in the instrument software were obtained from the raw intensity autocorrelation function of at least five different repetitions. The copolymers were dissolved in HPLC-grade water, chloroform, tetrahydrofuran, and dimethylformamide of (0.2 μm filtered). 

Small-angle X-ray scattering (SAXS) was executed via an Anton Paar (Graz, Austria) SAXSess system with a temperature-controlled sample cell with a quartz capillary (1 mm diameter, 10 μm wall thickness), sealed by vacuum-tight screwcaps on both ends. The vacuum during the measurements was ≈ 1 mbar. The solvent background contribution to the scattering intensity was recorded separately and subtracted on a relative scale (after normalization to the same transmissions) from all the samples SAXS intensities (20 g L^−1^ copolymer solutions to allow adequate scattering intensity for analysis). The Debyeflex 3003 (GE-Inspections technologies, Frankfurt, Germany) X-ray generator source was operated at 40 kV and 50 mA with a sealed-tube Cu anode (Cu-K𝛼 radiation source, 𝜆 = 0.154 nm). The X-ray beam was Goebel-mirror-focused and Kratky(line)-slit collimated with a final rectangular shaped (17 mm × 0.25 mm). A 1D MYTHEN-1k microstrip solid-state detector recorded the spectra in transmission mode, with a magnitude of the scattering vector *q* between 0.01 to 5 nm^−1^. Given 2𝜃 as the scattering angle (with respect to the incident beam) and 𝜆 as the wavelength of the X-rays, the sample to detector distance of 307 mm corresponded to a total 2𝜃 region of 0.14°–7°, applying the conversion *q* = 4𝜋 sin(𝜃)/𝜆. The exposure time was typically 60 s times 5, with a waiting time of 10 min between the different temperature steps for all spectra. 

## 3. Results and Discussion

### 3.1. Synthesis of PEGMA-co-SiMAx and TEGMA-co-SiMAx Copolymers 

**Figure 1:**
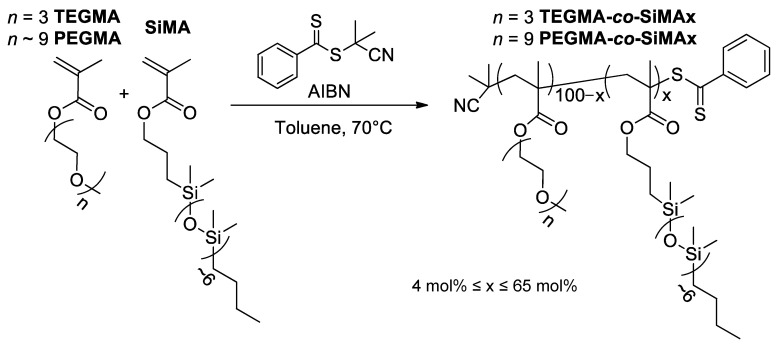
Synthesis of amphiphilic copolymers via RAFT polymerization.

The two sets of PEGMA-*co*-SiMAx and TEGMA-*co*-SiMAx amphiphilic copolymers contained the same hydrophobic (though not lipophobic) polydimethylsiloxane methacrylate (SiMA, *M*_n_ = 680 g/mol, number average degree of polymerization *n* ~ 6) component and hydrophilic poly(ethylene glycol) methyl ether methacrylate (PEGMA, *M*_n_ = 475 g/mol, number average degree of polymerization *n* ~ 9) or triethyleneglycol methyl ether methacrylate (TEGMA, *n* = 3) (Figure 1).

For both sets of copolymers, a reversible addition-fragmentation chain-transfer (RAFT) polymerization was chosen which employed 2-cyano-2-propyl benzodithioate as chain transfer agent (CTA) (Figure 1). The aromatic group in this CTA was easily detectable by ^1^H NMR, which is useful for the determination of the molecular weight and degree of polymerization of the different copolymer samples. The molar ratio between the two comonomers in the feed mixture was varied over a rather large composition range (5–70 mol% SiMA) (Table S1).

The RAFT polymerization was carried out with toluene as solvent at 70 °C under nitrogen, using 2,2′-azobis(2-methylpropionitrile) (AIBN) as the thermal initiator with a CTA:AIBN molar ratio of 5:1 for all the copolymerization runs. The reaction products were named PEGMA-*co*-SiMAx and TEGMA-*co*-SiMAx, where *x*, the molar percentage of SiMA, was determined by ^1^H NMR analysis, as well as the overall composition of the copolymer (Figures S1 and S2). The integral areas of the signals at 7.4–8 ppm of the aromatic protons in the CTA, 0.1 ppm for Si(CH_3_)_2_ protons of SiMA, 3.4 ppm for OCH_3_ protons of PEGMA or TEGMA, and 4–4.5 ppm for COOCH_2_ protons of both repeating units were used to calculate the number average degree of polymerization of the copolymers; the compositions are reported in Table 1. 

**Figure 2:**
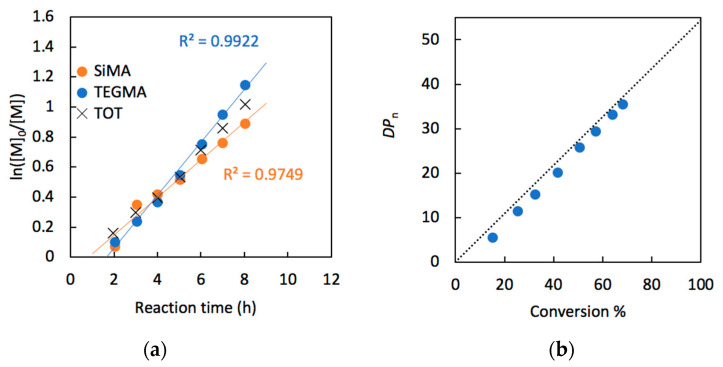
(**a**) Kinetics plot of SiMA, TEGMA and the sum of the two comonomers and (**b**) overall degree of polymerization (*DP*_n_, by ^1^H NMR in CDCl_3_) for the RAFT copolymerization (Monomers:CTA:AIBN = 55:1:0.2) of TEGMA and SiMA (50:50 molar ratio).

The controlled nature of the RAFT polymerization was verified by monitoring the kinetics of copolymer formation by ^1^H NMR, for the copolymerization of TEGMA and SiMA in 50:50 molar ratio. Figure 2a shows the kinetic plots of ln([*M*]_0_/[*M*]) vs. time, linear up to 8 h of polymerization where the total monomer conversion reached 64%. This implies a first-order kinetics of the consumption of monomers during the polymerization [50]. The plot also showed an induction time of about two hours, consistent with what already reported for polymerization mediated by the dithiobenzoate CTA used in this reaction [51,52]. As expected for a controlled RAFT polymerization, the number average degree of polymerization *DP_n_* grew linearly with conversion (Figure 2b), in agreement with the theoretical values given by Equation (1):

*DP_n_* = *p* [*M*]_0_/[*CTA*]_0_ (1)

where *p* is the monomer conversion and [*M*]_0_ and [*CTA*]_0_ are the initial molar concentrations of both comonomers and chain transfer agent, respectively.

GPC analysis confirmed the occurred copolymerization, and the copolymer curves were monomodal with a generally relatively low dispersity *Đ* ≤ 1.33, apart from copolymers PEGMA-*co*-SiMAx with a higher mole content (≥29%) of SiMA. The discrepancies in the *M_n_* determined by GPC and ^1^H NMR analysis were due to the calibration of the GPC setup with PMMA standards, that are characterized by a dissimilar hydrodynamic and conformational behavior, compared to the atypical one of amphiphilic copolymers. 

In keeping with the precise control of the macromolecular structure achieved by the RAFT copolymerization, the chemical composition of the copolymers was modulated over a predetermined wide molar range of both counits. This in turn enabled the production of several narrowly dispersed copolymers featuring diverse amphiphilic characters for investigation of self-assembling in solutions. While a few examples of amphiphilic copolymers based on fluorinated comonomers are reported in the literature [14,15,42,53,54,55], very little is known about the thermoresponsiveness and self-assembly in the water of amphiphilic copolymers based on siloxane comonomers [56].

### 3.2. Differential Scanning Calorimetry

**Figure 3:**
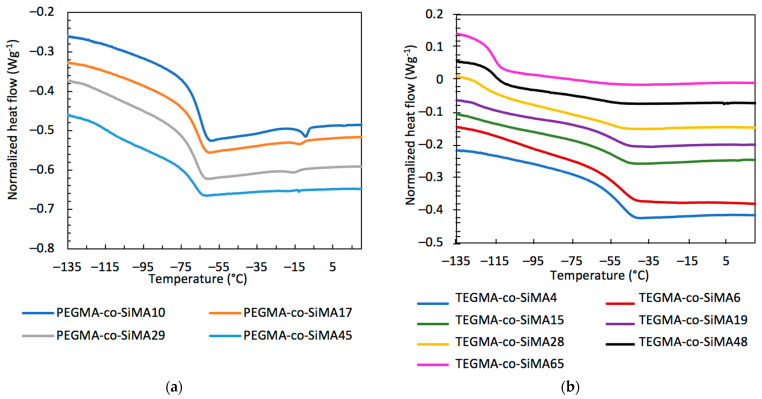
DSC second heating curves (exo^) of the copolymers PEGMA-*co*-SiMAx (**a**) and TEGMA-*co*-SiMAx (**b**).

Thermal characterization of synthesized copolymers was performed by differential scanning calorimetry (DSC) analyses (heating/cooling rates 10 °C min^−1^) with the objectives of identifying the glass transition temperature (*T*_g_) and its specific heat capacity change (Δ*C*_p_) as well as the melting temperature (*T*_m_) with its enthalpy variation (Δ*H*_m_). All samples were examined between −130 and 100 °C. Copolymers PEGMA-*co*-SiMAx were semicrystalline, whereas copolymers TEGMA-*co*-SiMAx were completely amorphous (Figure 3). For both copolymer systems, the overall thermal behavior depended on chemical composition.

The temperatures of glass and melting transitions and the associated specific heat and enthalpy changes are reported in Table 2. For copolymers with low amounts of SiMA, only one glass transition was detected similar to those of the homopolymers, p(PEGMA) (*T*_g_ = −63 °C) and p(TEGMA) (*T*_g_ = −48 °C). The copolymers with higher SiMA contents (≥45 mol% and 15 mol% for PEGMA-*co*-SiMAx and TEGMA-*co*-SiMAx, respectively) presented a second glass transition in the range −124 °C ÷ −115 °C that is similar to the behavior of the p(SiMA) homopolymer (*T*_g_ = −107 °C). This result suggests that the hydrophilic and the hydrophobic components were chemically incompatible and underwent microphase separation in the bulk. Moreover, a melting transition was also detected for PEGMA-*co*-SiMAx associated with the crystalline regions of PEGMA counits. Such a transition did not occur for TEGMA-*co*-SiMAx copolymers, given the shorter length of the oxyethylenic side chains, which is consistent with the completely amorphous nature of the corresponding homopolymer. The melting temperature was found to decrease from −9 °C to −18 °C as the content of PEGMA in the copolymer decreased from 90 to 55 mol% with a reduction in the melting enthalpy. The decrease in the crystallinity degree was due to the incorporation of SiMA counits as defective elements. 

### 3.3. Self-Assembly in Solution

#### 3.3.1. Light Transmittance Measurements

**Figure 4:**
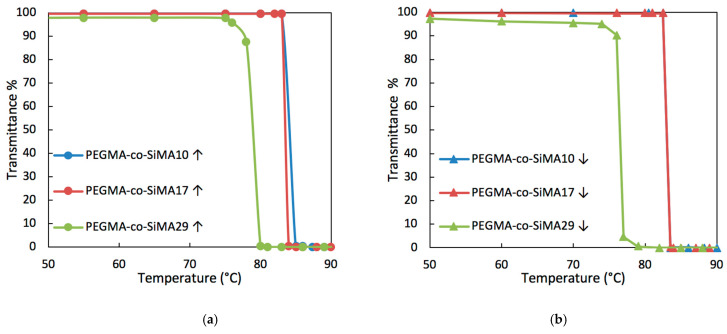

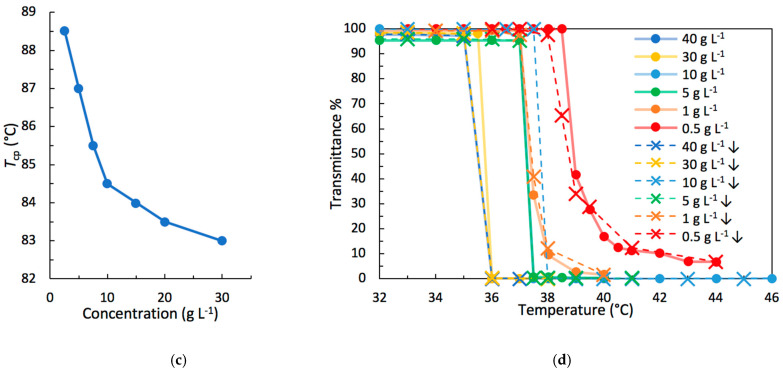
Light transmittance (𝜆 = 700 nm) vs. temperature for PEGMA-*co*-SiMAx copolymer solutions in water (10 g L^−1^) during heating (**a**) and cooling (**b**) ramp. *T*_cp_ as a function of copolymer concentration (2.5–30 g L^−1^) in water for PEGMA-*co*-SiMA10 (**c**). Light transmittance (𝜆 = 700 nm) vs. temperature for TEGMA-*co*-SiMA6 copolymer solutions in water with various concentrations in heating and cooling ramp (**d**).

Transmittance at a wavelength of *λ* = 700 nm of PEGMA-*co*-SiMAx and TEGMA-*co*-SiMAx solution in water was measured using a UV–vis spectrophotometer, thermostated (±0.1 °C) at different temperatures. Copolymers TEGMA-*co*-SiMAx with a SiMA content greater than 19 mol% were poorly soluble or completely insoluble in water and could not be investigated. The light transmittance values of solutions of PEGMA-*co*-SiMAx (with x = 10, 17, and 29 mol%) in water (10 g L^−1^) were plotted in Figure 4a,b as a function of temperature in a heating and cooling cycle, respectively. 

The curves in Figure 4a show a sharp reduction in the transmittance values from about 100% to 0% on heating to a critical temperature, named the cloud point temperature (*T*_cp_), at which copolymer aggregation into large multichain aggregates occurred. This phenomenon was also easily visually detected, since at room temperature the polymeric solution was transparent and homogeneous, whereas it turned into a cloudy dispersion at and above *T*_cp_. A cooling ramp was also carried out to evaluate the effective reversibility of the transition process (Figure 4b). For the solutions of copolymers with a SiMA content lower than 17 mol%, the transition was reversible with negligible hysteresis, whereas for the other samples, there was a slight thermal hysteresis by about 6–8 °C.

The *T*_cp_ was found to strongly depend on the type of hydrophilic component and its content in the copolymer. In particular, for both sets of copolymers, *T*_cp_ increased as the content of the hydrophilic component increased, tending to the values of the respective homopolymers pPEGMA (*T*_cp_ = 90 °C) and pTEGMA (*T*_cp_ = 47 °C) [59]. This result confirms that the amphiphilic copolymers of this work showed a similar thermoresponsive behavior as their respective homopolymers, by which hydrogen bonds between water and hydrophilic oxyethylene side chains are broken at *T*_cp_ and intermacromolecular polar bonds among copolymer chains are formed, thereby giving rise to multichain aggregates. Moreover, for a similar content of the hydrophilic component in the copolymer, the *T*_cp_ decreased by more than 50 °C (copolymer concentration 10 g L^−1^) by passing from PEGMA to TEGMA counits (Table 3). According to the literature [4,59], the longer oxyethylene side chains guarantee a higher hydrophilicity and assist water solubility of the non-aggregated (co)polymer chains in the aqueous medium over an extended temperature interval.

To investigate the influence of the concentration on the thermoresponsive behavior, aqueous solutions of selected copolymers were prepared at various concentrations and analyzed through transmittance measurements by varying the temperature (Table 3). By increasing the concentration of the copolymer, a reduction in the value of *T*_cp_ was observed down to a plateau, consistent with a lower critical solution temperature (LCST)-type behavior (Figure 4c,d).

#### 3.3.2. Dynamic Light Scattering

Dynamic light scattering (DLS) measurements were carried out on the copolymers PEGMA-*co*-SiMAx and TEGMA-*co*-SiMAx to investigate the ability of these amphiphilic copolymers to form self-assembled nanostructures in water. Furthermore, the influence of temperature on these systems was assessed. For each PEGMA-*co*-SiMAx water-soluble sample, aqueous solutions with a concentration of 10 g L^−1^ of copolymer were analyzed by DLS varying the temperature in the range 25–90 °C. On the other hand, copolymers TEGMA-*co*-SiMAx were analyzed at a reduced concentration of 1 g L^−1^ to minimize the turbidity of the solution. Copolymers with a SiMA content higher than 19 mol% were not analyzed because of their complete insolubility. The hydrodynamic diameter (*D*_h_) values at room temperature and at a temperature above *T*_cp_ are collected in Table 4 for the two series of copolymers. 

**Figure 5:**
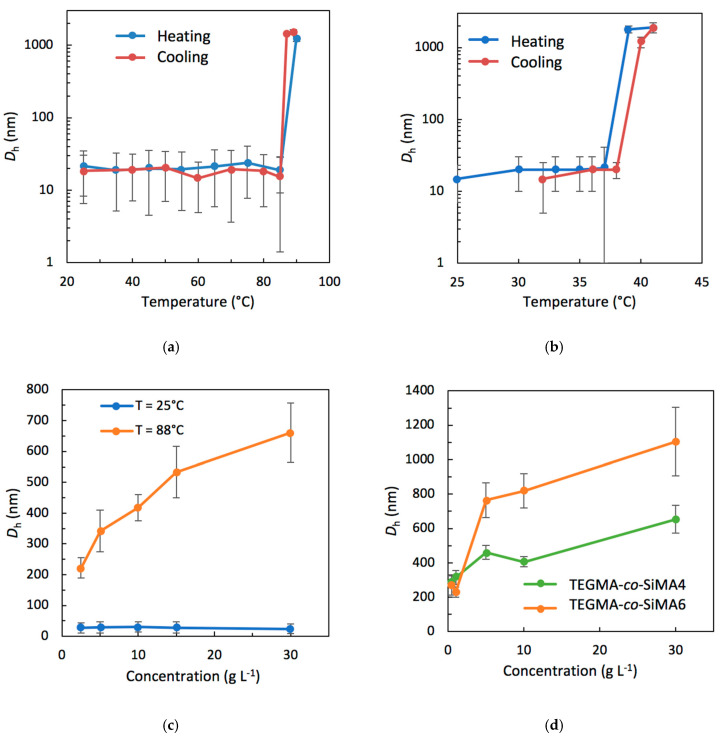
Variation of the hydrodynamic diameters (*D*_h_) as a function of temperature in a heating and cooling cycle for aqueous solutions (10 g L^−1^) of PEGMA-*co*-SiMA10 (**a**) and TEGMA-*co*-SiMA6 (**b**). Hydrodynamic diameters (*D*_h_) of PEGMA-*co*-SiMA29 at 88 °C (*T* > *T*_cp_) as a function of concentration. (**c**). Hydrodynamic diameters (*D*_h_) of TEGMA-*co*-SiMA4 at 60 °C (*T* > *T*_cp_) and TEGMA-*co*-SiMA6 at 45 °C (*T* > *T*_cp_) as a function of concentration (**d**).

The DLS measurements of the PEGMA90-*co*-SiMA10 and TEGMA-*co*-SiMA6 copolymers will be described as a typical example (Figure 5a and Figure 5b, respectively). A drastic sharp increase in *D*_h_ at a temperature above *T*_cp_ was observed, confirming the light transmittance results (Figure 4). In fact, the cloud point observed could be related to an abrupt change in the aggregation state of the amphiphilic copolymers in water. This finding was attributed to the formation of smaller micellar nanostructures at *T* < *T*_cp_ and larger aggregates at *T* ≥ *T*_cp_. The *D*_h_ values measured in a cooling cycle were similar to those obtained during heating, confirming the reversibility of the thermoresponsive phenomenon. 

For any copolymer solution investigated herein (Table 4), we observed that increasing the SiMA mole content in the copolymer resulted in an increase in the size of the nanostructures observed at *T < T*_cp_, and a decrease in the *D*_h_ measured at *T > T*_cp_ up to the point reached for the copolymer PEGMA-*co*-SiMA45, for which the difference between the two values was the smallest one (30 nm).

The effect of copolymer concentration on self-assembly was also tested. DLS measurements were carried out by varying the concentration (from 0.5 to 30 g L^−1^) of copolymer in the water at 25 °C and at *T* > *T*_cp_. The case of PEGMA-*co*-SiMA29 will be discussed in detail as an example (Figure 5c). These analyses showed that at a temperature below *T*_cp_, the *D*_h_ of the nanostructures did not change significantly with the increase in concentration of the copolymer in the water solution, with a behavior that is compatible with a micellar, close aggregation mechanism, for the concentration range investigated. Differently, at a temperature above *T*_cp_, the micellar nanoparticles collapse into larger aggregates that showed an open aggregation mechanism, with an increase in the size of the aggregate at higher copolymer concentrations (Figure 5c,d).

**Figure 6:**
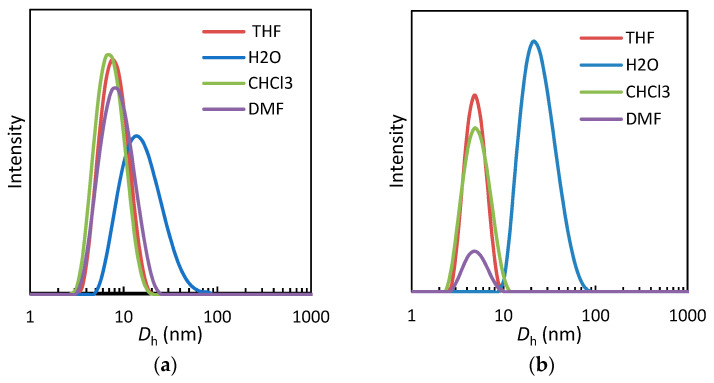
DLS intensity size distribution of hydrodynamic diameters (*D*_h_) for PEGMA-*co*-SiMA10 (**a**) and TEGMA-*co*-SiMA15 (**b**) copolymers in different solvents at 25 °C.

The effect of organic solvents on the self-assembly of the amphiphilic copolymers at room temperature was also investigated. Chloroform, tetrahydrofuran (THF) and dimethylformamide (DMF) were used as solvents with lower selectivity than water towards the hydrophilic/hydrophobic component of the copolymers. Measured average hydrodynamic diameters are reported in Table 4. As an example, in Figure 6a, the intensity size distribution was also compared with that in water for copolymer PEGMA-*co*-SiMA10. Particle size in organic solvents was generally found to be 5 nm ≤ *D*_h_ ≤ 15 nm, that was significantly smaller than in water. The hydrodynamic diameter of the nanostructures in aqueous solution increased with increasing SiMA content in the copolymer, going from 17 nm to 71 nm for the PEGMA-*co*-SiMA series and from 13 nm to 37 nm for the TEGMA-*co*-SiMA series. The relatively large sizes of the nanostructures formed in aqueous solutions at *T* < *T*_cp_ seem to be compatible with the formation of multichain micellar nanoparticles rather than single-chain, unimer micelles that are normally characterized by *D*_h_ < 10 nm [15,60]. In agreement with the results from SAXS analysis (see below Section 3.3.3) we assume that in the tested organic solvents the copolymers were totally solvated, assuming a typical random coil conformation. In contrast, in aqueous solution, the polymer chains self-assembled into micellar nanoparticles due to the favorable interactions of water with oxyethylenic side chains and hydrophobic interaction among the siloxane ones. This assembly resulted in distinct hydrophobic compartments whose non-polar nature was confirmed by the solvatochromic shift of CTA residue absorption in UV–vis spectra (see Section 3.3.4 below).

#### 3.3.3. Small-Angle X-ray Scattering

**Figure 7:**
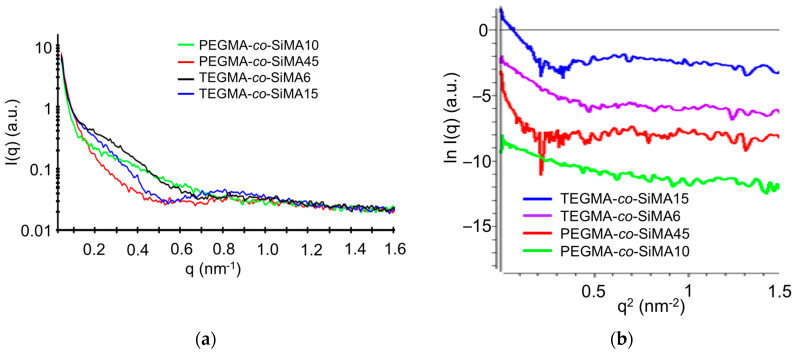
SAXS intensity profiles for background-subtracted water solutions of copolymers (20 g L^−1^) PEGMA-*co*-SiMAx and TEGMA-*co*-SiMAx at 25 °C (**a**). Guinier plots of water solutions of copolymers (20 g L^−1^) (**b**). Plots are shifted vertically by a constant factor for better visibility.

To investigate the morphology of the micellar nanoparticles in solution, some of the synthesized amphiphilic copolymers were analyzed by small-angle X-ray scattering (SAXS) measurements. In particular, aqueous and THF solutions (20 g L^−1^) of copolymers PEGMA-*co*-SiMA10, PEGMA-*co*-SiMA45, TEGMA-*co*-SiMA6 and TEGMA-*co*-SiMA15 were analyzed by SAXS. In Figure 7a we report the scattering intensity *I*(*q*) of all samples in water as a function of the scattering vector *q* after background subtraction. 

The Guinier plots (Figure 7b), linear in the region of low *q* values (1/*R*_g_ < *q* < 1.3/*R*_g_), were used to determine the radius of gyration *R*_g_ (Table 5) of the particles. In the water solution, the Guinier curves showed a positive variation with respect to linearity at low *q* values (*q*^2^ < 0.4 nm^–2^). This deviation suggests the presence of attractive interactions between the molecules in aqueous solution, confirming the formation of multichain micellar nanoparticles. Additionally, *R*_g_ values calculated for all the samples in water were higher than those in THF, in agreement with what was shown by the DLS measurements. The combined SAXS and DLS results suggest that these copolymers self-assembled in water solution in small aggregates due to the intermacromolecular interactions among copolymer chains.

**Figure 8:**
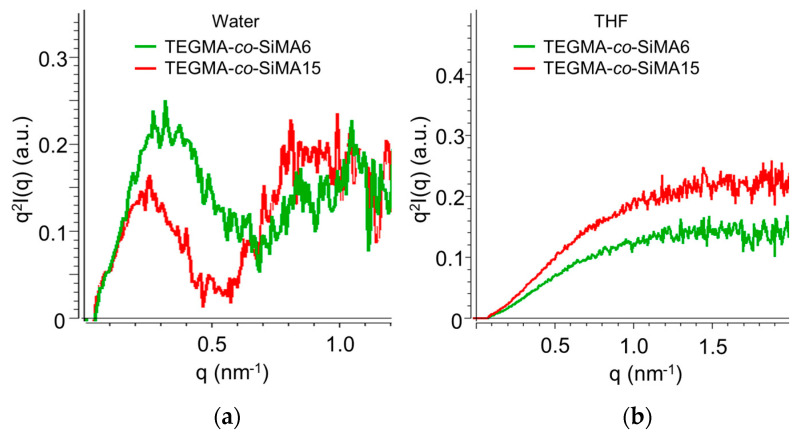
Kratky plots for copolymer TEGMA-*co*-SiMAx in water (**a**) and THF (**b**).

Kratky plots (*q*^2^*I*(*q*) vs. *q*) of copolymer solutions in water (selective solvent) and THF (non-selective solvent) are compared in Figure 8. TEGMA-*co*-SiMA6 and TEGMA-*co*-SiMA15 displayed in THF (Figure 8b) ascending *q*^2^*I*(*q*) curves until a plateau was reached in the high *q* region, typical of polymers that adopt a random coil conformation. By contrast, Kratky plots of the copolymer in water (Figure 8a) showed a small peak followed by a large shoulder at higher *q*. This shape of the plot indicates the partial degree of folding within the nanostructures, due to the hydrophobic interactions among the hydrophobic portions of the copolymer such as the siloxane chains. Moreover, the real space *p*(*r*)-function was obtained via indirect Fourier transformation [61] of the scattering curve *I*(*q*) in reciprocal space. This kind of plot is also called distance distribution function and helps to elucidate the shape and conformation of polymer particles [62,63].

**Figure 9:**
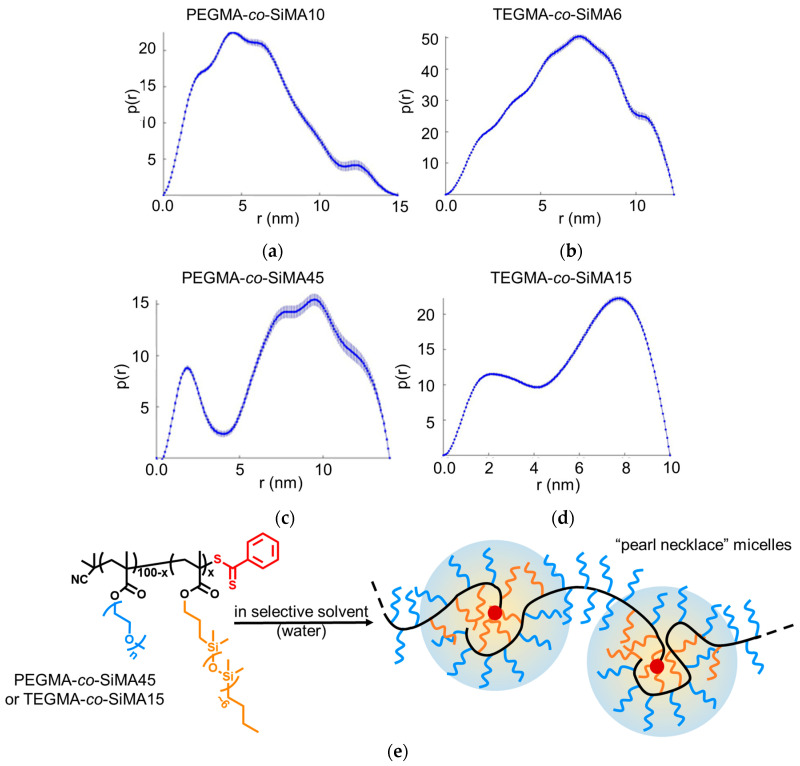
Distance distribution *p*(*r*)-function of water solutions (20 g L^−1^) of copolymers PEGMA-*co*-SiMAx and TEGMA-*co*-SiMAx (**a**–**d**). Schematic representation of the self-assembly in “pearl necklace” micelles of the copolymers with higher SiMA content (**e**).

Figure 9 demonstrates that the maximum dimension of the copolymer particle in water solution was in any case on the order 10–15 nm. Moreover, for PEGMA-*co*-SiMA10 and TEGMA-*co*-SiMA6 the maximum of the *p*(*r*)-function correlated well with the *R*_g_ values obtained from the Guinier analysis. However, the asymmetric profile of the curve as well as the presence of several humps (Figure 9a,b) were indicative of an anisotropic shape of the non-homogeneous and non-compact aggregates [64]. On the other hand, the *p*(*r*)-function plot for the copolymers richer in SiMA (PEGMA-*co*-SiMA45 and TEGMA-*co*-SiMA15) (Figure 9c,d) more clearly indicated the presence of multicore, pearl necklace micelles in solution. For these samples, a first peak (*r* ≈ 2 nm) correlates to the diameter of one globule slightly smaller than the size of a single polymer chain in THF (*R*_g_ ≈ 2−2.3 nm); a second peak (*r* ≈ 8–10 nm) arises from the pairing distances between neighboring globules [65,66]. There are also larger structures present which could not be resolved well with our SAXS measurements as they were beyond our resolution and which are the reason for the sharp cutoff of the *p*(*r*)-functions at *r* > 10 and *r* > 12 nm, respectively, in Figure 9b–d.

Thus, the pearl necklace structure (Figure 9e) seemed to acquire greater dimensional uniformity and conformational homogeneity with the increase in SiMA content in the copolymer. Increasing the percentage of such a component could lead to the formation of more compact, folded domains within the single polymer chain arising from the stronger hydrophobic interactions of SiMA side chains. Such folded domains are connected by unfolded flexible portions. Differently, copolymers richer in PEGMA, being overall more soluble in water were less susceptible to fold intramolecularly in compact nanostructures to shield the hydrophobic compartment, thus aggregating structurally less-defined nanoparticles. Necklace-like micelle morphology was recently reported in the literature to describe the aggregation in water of amphiphilic copolymers of PEG-acrylamide and dodecyl acrylamide [23]. It is also reported for other systems such as copolymers of sodium maleate and dodecyl vinyl ether [7,67,68,69].

#### 3.3.4. Solvatochromism of the RAFT CTA 

**Figure 10:**
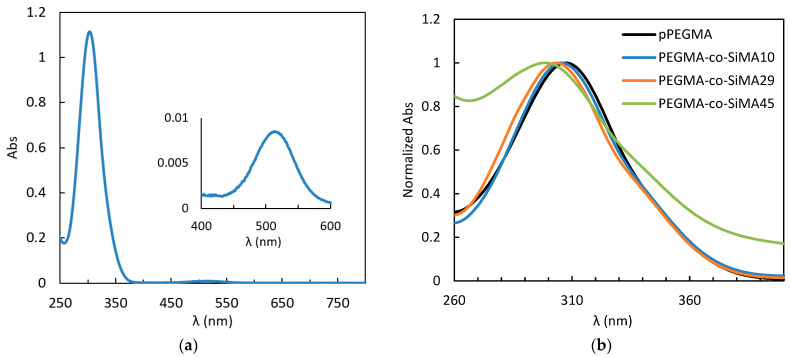
UV–vis spectra of CTA 10^−4^ M in CHCl_3_ (**a**) and the series of PEGMA-*co*-SiMAx copolymers in water compared to the homopolymer pPEGMA at 10^–4^ M (or 4 × 10^–5^ M when SiMA > 29 mol%) (**b**).

The UV–vis spectrum of the RAFT chain transfer agent used is characterized by a maximum of absorption at *λ*_max_ = 303 nm and a second minor peak at *λ* = 521 nm in CHCl_3_ as shown in Figure 10a. Moreover, *λ*_max_ showed solvatochromic behavior, with a bathochromic shift of ~10 nm from 298 nm to 308 nm with increasing solvent polarity from *n*-hexane/PDMS to water (Table 6).

Thus, the residue of the CTA attached to the macromolecular chain end is also anticipated to be sensitive to the nano-environment derived from the self-assembly of the copolymers in water. In fact, the CTA residue in pPEGMA showed a *λ*_max_ in water solutions similar to those in water itself (307 nm), but a decrease in *λ*_max_ was detected for water solutions of the amphiphilic copolymers. In particular, *λ*_max_ was found to decrease by increasing the SiMA content in the copolymer, reaching a minimum value of 299 nm for PEGMA-*co*-SiMA45 (Figure 10b, Table 6). This finding demonstrates that the CTA residue is exposed to an environment characterized by a polarity comparable to that of *n*-hexane and PDMS. This is consistent with the preferential formation of more compact folded domains with hydrophobic siloxane core shielded by hydrophilic PEG chains (Figure 9e). This was also showed by SAXS analysis by copolymer with higher SiMA contents.

## 4. Conclusions

PEGMA (or TEGMA)-*co*-SiMAx amphiphilic copolymers with modulated solubility in water, were synthesized by RAFT controlled polymerization. All water-soluble copolymers demonstrated reversible LCST-like thermoresponsive features, typically with a cloud-point temperature *T*_cp_ which was predominantly dependent on the hydrophilic oligo(ethylene glycol) side chain length and the percentage of the hydrophobic SiMA comonomer. By easily varying these structural parameters, the *T*_cp_ of copolymer water solutions (10 g L^–1^) was tailored to span a wide range (from ~85 °C to ~27 °C), passing across the physiological temperature. Thermoresponsiveness was also affected by the concentration of the copolymer in solution. 

The copolymer nanostructures in water below *T*_cp_ were deeply characterized by DLS and SAXS. The *D*_h_ increased with the SiMA content, varying from 17 nm to 70 nm in going from PEGMA-*co*-SiMA10 to PEGMA-*co*-SiMA45. These dimensions suggested the formation of multichain micellar nanostructures whose multicore pearl necklace-like morphology was clearly proved by SAXS for both PEGMA and TEGMA-based copolymers richer in SiMA counits. By contrast, copolymers showed a random coil conformation in THF, given the non-selective nature of this solvent as opposite to water. The covalently linked RAFT CTA moiety exhibited a solvatochromic effect as a result of its preferential compartmentalization in the apolar environment typical of the hydrophobic cores of the micelles in water. 

In conclusion, these amphiphilic copolymers, owing to simple chemical modification, were susceptible to modulations in their nano-assembling capacity and responses to external stimuli. In particular, the copolymers of the series TEGMA-*co*-SiMAx, with the shorter hydrophilic side chain and characterized by the lower *T*_cp_ range (41–27 °C), are the most promising for application as thermoresponsive carriers with a trigger across the human physiological temperature. In several examples, the dimension of such nano-assemblies was in the sub-100 nm realm. These features make them worth investigating as smart carriers for the encapsulation and delivery of hydrophobic molecules, especially drugs and bioimaging agents. A gradual increase in the hydrophobic character of the micelle compartments was observed by increasing the SiMA content in the copolymer; thus, we can speculate that different micelles acting as carriers could accommodate functional molecules with different degrees of hydrophobicity.

## Figures and Tables

**Table 1:** Physical–chemical characterization of copolymers PEGMA-*co*-SIMAx and TEGMA-*co*-SiMAx.

Copolymer	Conversion ^(a)^ (%)	SiMA (mol%)	SiMA (wt%)	*M*_n_^(b)^ (g mol^–1^)	*M*_n_^(c)^ (g mol^–1^)	*Đ* ^(c)^	Water Solubility
PEGMA-*co*-SiMA10	97	10	14	20,300	17,300	1.30	Yes
PEGMA-*co*-SiMA17	96	17	23	23,500	21,000	1.29	Yes
PEGMA-*co*-SiMA29	94	29	37	31,500	17,600	1.59	Yes
PEGMA-*co*-SiMA45	74	45	54	29,000	15,600	1.60	Yes
TEGMA-*co*-SiMA4	96	4	12	7700	17,100	1.17	Yes
TEGMA-*co*-SiMA6	97	6	17	8500	16,100	1.16	Yes
TEGMA-*co*-SiMA15	94	15	34	8000	20,000	1.22	Yes
TEGMA-*co*-SiMA19	89	19	41	8700	21,400	1.17	Yes
TEGMA-*co*-SiMA28	90	28	53	8800	20,400	1.13	No
TEGMA-*co*-SiMA48	80	48	73	12,200	26,000	1.33	No
TEGMA-*co*-SiMA65	78	65	85	8600	22,500	1.30	No

^(a)^ Total conversion of the monomers in the crude product determined by ^1^HNMR. ^(b)^ Number average molecular weight by ^1^HNMR. ^(c)^ Number average molecular and dispersity by GPC.

**Table 2:** Thermal properties of the copolymers PEGMA-*co*-SiMAx and TEGMA-*co*-SiMAx.

Copolymer	*T*_g_ ^(a)^ (°C)	Δ*C*_p_ ^(a)^ (J (gK)^−1^)	*T*_g_ ^(b)^ (°C)	Δ*C*_p_ ^(b)^ (J (gK)^−1^)	*T*_m_ ^(b)^ (°C)	Δ*H*_m_ ^(b)^ (J g^−1^)
pPEGMA^(c)^			−63	1.44		
PEGMA-*co*-SiMA10	n.d. ^(d)^		−64	1.20	−9	−0.64
PEGMA-*co*-SiMA17	n.d. ^(d)^		−66	0.86	−12	−0.23
PEGMA-*co*-SiMA29	n.d. ^(d)^		−67	0.78	−15	−0.26
PEGMA-*co*-SiMA45	−120	0.23	−68	0.45	−18	−0.06
pTEGMA ^(c)^			−48	0.60		
TEGMA-*co*-SiMA4	n.d. ^(d)^		−50	0.63		
TEGMA-*co*-SiMA6	n.d. ^(d)^		−49	0.59		
TEGMA-*co*-SiMA15	−122	0.09	−53	0.34		
TEGMA-*co*-SiMA19	−124	0.19	−52	0.32		
TEGMA-*co*-SiMA28	−123	0.29	−53	0.21		
TEGMA-*co*-SiMA48	−115	0.38	−58	0.11		
TEGMA-*co*-SiMA65	−115	0.55	n.d. ^(d)^			
pSiMA ^(c)^	−107	0.95				

^(a)^ Glass transition temperature and specific heat capacity change for the SiMA component. ^(b)^ Glass transition temperature, specific heat capacity change, melting temperature and enthalpy for the PEGMA or TEGMA component. ^(c)^ Data from the literature [15,57,58]. ^(d)^ Not determined.

**Table 3:** Cloud temperature of copolymer solutions in water at different concentrations (error on *T*_cp_ within ± 0.2 °C).

Copolymer	Concentration *(*g L^−1^*)*	*T*_cp_ (°C)
PEGMA-*co*-SiMA10	2.5	88.5
	5.0	87.0
	7.5	85.5
	10	84.5
	15	84.0
	20	83.5
	30	83.0
PEGMA-*co*-SiMA17	10	83.0
PEGMA-*co*-SiMA29	10	79.0
PEGMA-*co*-SiMA45	10	73.0
TEGMA-*co*-SiMA4	10	41.0
TEGMA-*co*-SiMA6	0.5	38.9
	1.0	37.3
	5.0	37.3
	10	37.2
	30	35.8
	40	35.5
TEGMA-*co*-SiMA15	0.5	33.0
	1.0	31.0
	5.0	28.5
	10	28.4
	40	28.3
TEGMA-*co*-SiMA19	0.5	33.0
	1.0	32.0
	5.0	29.5
	10	26.5

**Table 4:** Average hydrodynamic diameters (*D*_h_) by DLS of copolymer solutions in various organic solvents (20 g L^−1^) and water (10 g L^−1^) at 25 °C and above *T*_cp_ for PEGMA-*co*-SiMAx and TEGMA-*co*-SiMAx.

Copolymer	*D*_h_ (H_2_O) (nm)		*D*_h_ (CHCl_3_) (nm)	*D*_h_ (DMF) (nm)	*D*_h_ (THF) (nm)
	25 °C	*T* > *T*_cp_	25 °C	25 °C	25 °C
PEGMA-*co*-SiMA10	17 ± 9	1200 ± 100 ^(a)^	7 ± 3	8 ± 3	8 ± 3
PEGMA-*co*-SiMA29	30 ± 20	420 ± 40 ^(a)^	11 ± 4	10 ± 4	9 ± 3
PEGMA-*co*-SiMA45	70 ± 40	100 ± 10 ^(a)^	10 ± 5	15 ± 6	9 ± 4
TEGMA-*co*-SiMA4	13 ± 4	310 ± 40 ^(b)^	5 ± 2	6 ± 1	5 ± 1
TEGMA-*co*-SiMA6	16 ± 4	230 ± 30 ^(b)^	8 ± 4	7 ± 3	11 ± 6
TEGMA-*co*-SiMA15	24 ± 9	140 ± 30 ^(b)^	5 ± 1	5 ± 1	5 ± 1
TEGMA-*co*-SiMA19	37 ± 9	170 ± 60 ^(b)^	5 ± 2	6 ± 2	5 ± 1

^(a)^ Measured at *T* > 85 °C and 10 g L^−1^. ^(b)^ Measured at *T* > 60 °C and 1 g L^−1^.

**Table 5:** Radius of gyration *R*_g_ of copolymer solutions in water and THF (20 g L^−1^) at 20 °C.

Copolymer	Solvent	Aggregation	*R*_g_ (nm)
PEGMA-*co*-SiMA10	H_2_O	Aggregates	5.0 ± 0.3
	THF	Random coil	2.30 ± 0.03
PEGMA-*co*-SiMA45	H_2_O	Aggregates	6.1 ± 0.1
	THF	Random coil	2.01 ± 0.07
TRIGMA-*co*-SiMA6	H_2_O	Aggregates	6.3 ± 0.3
	THF	Random coil	2.26 ± 0.05
TRIGMA-*co*-SiMA15	H_2_O	Aggregates	7.1 ± 0.6
	THF	Random coil	2.31 ± 0.04

**Table 6:** Wavelength of maximum absorption *λ*_max_ of CTA (10^–4^ M) in solvents with different relative permittivity (*ε*_r_) compared with the *λ*_max_ of the residue of the CTA of the amphiphilic copolymers in water solution.

CTA in Solvent	*λ*_max_ (nm)	*Ɛ*_r_ 20 °C	Copolymer in Water	*λ*_max_ (nm)
H_2_O	308	81.1	pPEGMA	307
DMSO	307	46.7	PEGMA-*co*-SiMA10	305
Diglyme	304	7.3	PEGMA-*co*-SiMA29	303
CH_2_Cl_2_	303	9.1	PEGMA-*co*-SiMA45	299
THF	303	7.5		
CHCl_3_	303	4.8	TEGMA-*co*-SiMA4	305
*n*-hexane	299	1.9	TEGMA-*co*-SiMA6	304
PDMS	298	2.6	TEGMA-*co*-SiMA15	303

## Data Availability

The data presented in this study are available on request.

## Supplementary Materials

The following supporting information can be downloaded at: https://www.mdpi.com/article/10.3390/pharmaceutics15061703/s1, Table S1: Reaction conditions for the RAFT synthesis of copolymers PEGMA-*co*-SIMAx and TEGMA-*co*-SiMAx. Figure S1: ^1^H NMR spectrum of PEGMA-*co*-SiMA45 in acetone-d6. Figure S2: ^1^H NMR spectrum of TEGMA-*co*-SiMA48 in acetone-d6.
