# Supplementary material for: The Nanostructured Self-Assembly and Thermoresponsiveness in Water of Amphiphilic Copolymers Carrying Oligoethylene Glycol and Polysiloxane Side Chains
Source: Pharmaceutics. 2023 Jun 10;15(6):1703. doi: 10.3390/pharmaceutics15061703 (PMC10301929; doi:10.3390/pharmaceutics15061703)
Supplement: Supplementary file 1 [file pharmaceutics-15-01703-s001.zip › pharmaceutics-2404649-supplementary.pdf]

Supporting information

Table S1. Reaction conditions for the RAFT synthesis of copolymers PEGMA-*co*-SIMAx and TEGMA-*co*-SiMAx.

Copolymer	PEGMA(TEGMA) mL (mmol)	SiMA mL (mmol)	Monomer ratio	AIBN mg (mmol)	CTA mg (mmol)	Toluene (mL)	Time (h)	Yield (%)
PEGMA-*co*-SiMA10	1.60 (3.49)	0.28 (0.39)	90:10	2.55 (0.015)	17.19 (0.077)	2.6	15	85
PEGMA-*co*-SiMA17	1.37 (2.99)	0.53 (0.75)	80:20	2.46 (0.015)	16.58 (0.075)	2.5	15	82
PEGMA-*co*-SiMA29	1.16 (2.53)	0.77 (1.08)	70:30	2.37 (0.014)	16.01 (0.072)	2.4	15	73
PEGMA-*co*-SiMA45	0.78 (1.69)	1.21 (1.69)	50:50	2.22 (0.013)	14.98 (0.068)	2.2	15	71
TEGMA-*co*-SiMA4	1.680 (7.45)	0.280 (0.39)	95:5	0.0053 (0.03)	0.0347 (0.15)	5.2	15	73
TEGMA-*co*-SiMA6	1.466 (6.48)	0.5147 (0.72)	90:10	0.0051 (0.03)	0.032 (0.14)	4.8	15	58
TEGMA-*co*-SiMA15	1.280 (5.66)	0.714 (1.00)	85:15	0.0045 (0.03)	0.0295 (0.13)	4.4	15	55
TEGMA-*co*-SiMA19	1.120 (4.95)	0.880 (1.23)	80:20	0.0042 (0.02)	0.0275 (0.12)	4.1	15	55
TEGMA-*co*-SiMA28	0.860 (3.80)	1.164 (1.63)	70:30	0.0035 (0.02)	0.0247 (0.11)	3.6	15	53
TEGMA-*co*-SiMA48	0.492 (2.18)	1.556 (2.18)	50:50	0.0029 (0.02)	0.0193 (0.09)	2.9	15	65
TEGMA-*co*-SiMA65	0.250 (1.09)	1.820 (2.54)	30:70	0.0026 (0.01)	0.0179 (0.07)	2.4	15	76

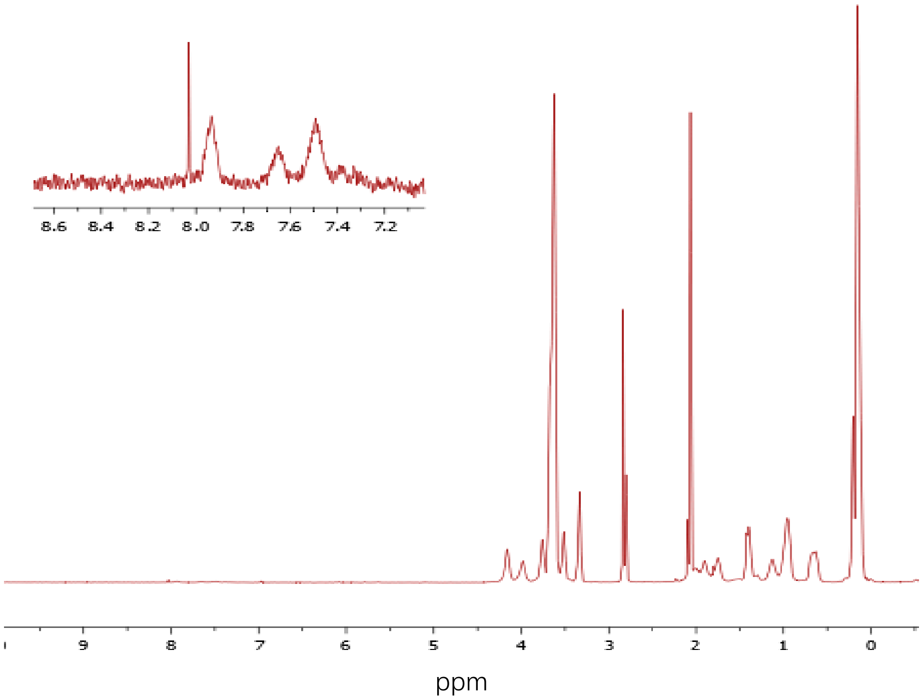

Figure S1. ^{1}H NMR spectrum of PEGMA-*co*-SiMA45 in acetone-d6.

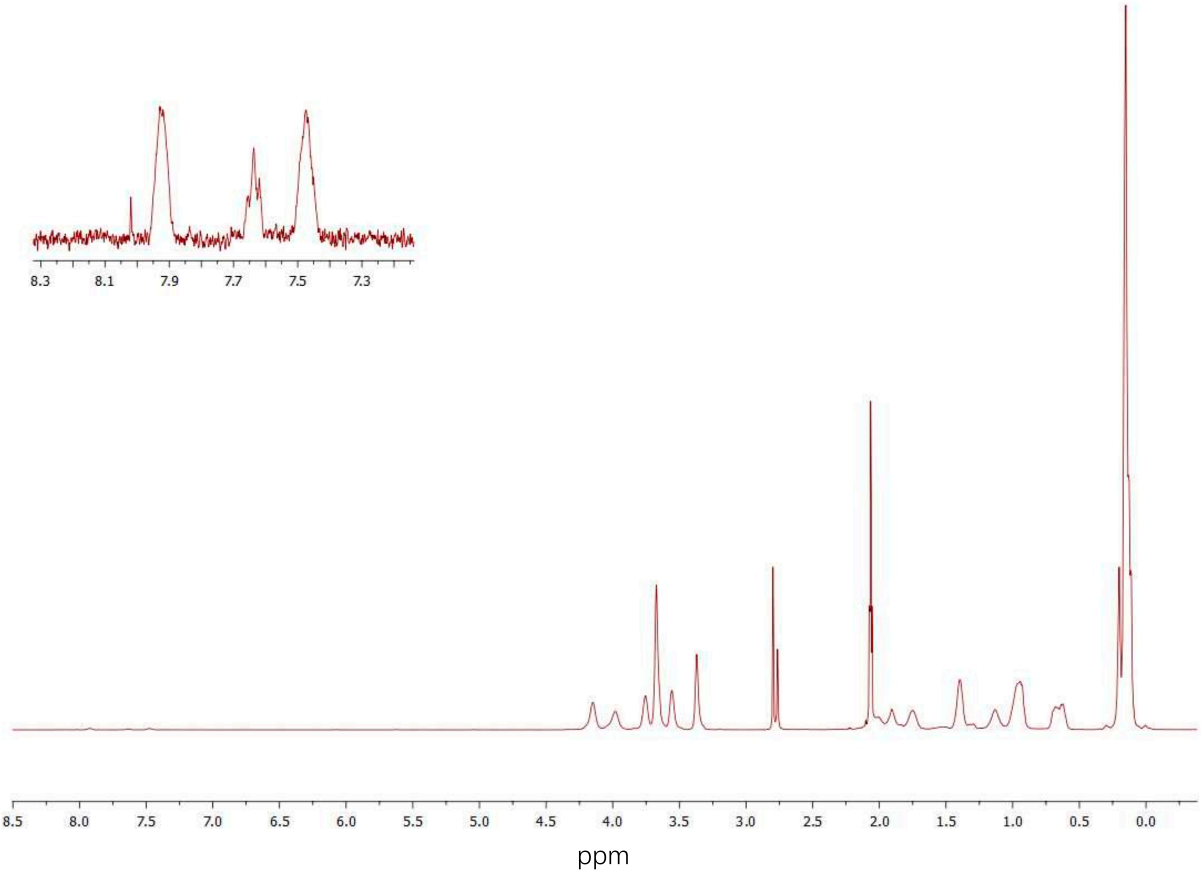

Figure S2. ^{1}H NMR spectrum of TEGMA-*co*-SiMA48 in acetone-d6.
